# Two Wire Sensor for Measuring the Velocity of Non-Isothermal Flows

**DOI:** 10.3390/s22010162

**Published:** 2021-12-27

**Authors:** Katarzyna Socha, Paweł Jamróz

**Affiliations:** Strata Mechanics Research Institute, Polish Academy of Sciences, Reymonta 27, 30-059 Krakow, Poland; jamroz@imgpan.pl

**Keywords:** correlation method, flow velocity measurement, temperature measurement

## Abstract

Changes in the temperature of the medium significantly affect the static characteristics of hot-wire anemometry measuring wires, which causes errors in the results of flow velocity measurements. High temperatures of the medium make it necessary to additionally heat the sensor to even higher temperatures, which may lead to its damage due to wire burnout. The article proposes a solution to the problem of measuring the flow velocity in conditions of non-stationary temperatures with the use of the method of cross-correlation of signals from two-wire resistance thermometers. The main assumptions of the method and its experimental verification were presented.

## 1. Introduction

Methods of measuring small values of flow velocities in the range up to 1 ms^−1^ are still being developed, and the measuring methods developed so far are characterized by large uncertainties resulting from the abilities and limitations of the constructed measuring instruments.

Hot-wire anemometry measurement methods allow us to determine the flow velocity using the changes in the intensity of the heat exchange of the heated wire with the environment, along with the change of the medium flow velocity [1,2]. At low speeds, due to the phenomena related to the occurrence of free convection from the heated element, the function that determines the relationship between the velocity of the medium and the heat transfer intensity becomes undefined. The application limit of the measurement method is estimated at 0.2–0.3 ms^−1^, depending on the type of sensor and the heating wire heating coefficient [1]. However, this method is very sensitive to changes of parameters related to the medium itself, in particular its temperature, pressure and humidity, which also change the static characteristics of the measuring sensor.

Based on the theory of thermal waves, a method of absolute measurement of gas flow velocity has been developed, which is used in velocity measurements in the range of 0.1 to 3 ms^−1^ [3,4]. The method uses three wires arranged in parallel to each other, located on one plane, one of which is heated periodically by changing the heating coefficient and constitutes the source of the heat wave. The next two wires are located in the thermal trace, and the distance between them is strictly defined. These wires measure the temperature wave generated by the first wire. Based on the analysis of the thermometer’s wires response, it is possible to determine the flow velocity based on the phase shift between the response signals of the thermometric sensors [5,6].

Both of the presented methods (hot-wire anemometry and thermal waves), allowing for the measurement of low velocities, have limitations as they cannot be used to measure flows in the conditions of high temperature fluctuations of hundreds of degrees Celsius. Measurement in such conditions would require heating the measuring wire or the one generating the temperature wave well above the temperature of the medium, which would damage the wire. Fluctuations of high temperature are a source of the errors for such measurement methods. It requires using compensation in the measurement process, which assumes an extra temperature sensor. Such a sensor should be able to measure temperature fluctuations with high bandwidth to provide reliable compensation [7,8].

Performing measurements in flows under high temperature conditions is important from the point of analysis of flows occurring, among others, in calibration furnaces, climatic test chambers, ventilation systems of electronic systems mounted on busbars [9] or annealing furnaces [10].

One method that allows for measurement velocity of non iso-thermal flows is a method assuming utilization of the two hot-wire anemometers. The velocity is calculated from the two hotwire anemometers operating at different overheat output signals [11,12]. This method is complex and not adapted to measurements in the range of high temperatures.

To analyze the phenomena, an attempt was made to develop a method for measuring low velocity values under conditions of non-isothermal flows.

## 2. Correlative Method of Measuring Flow Velocity

**Figure 1:**
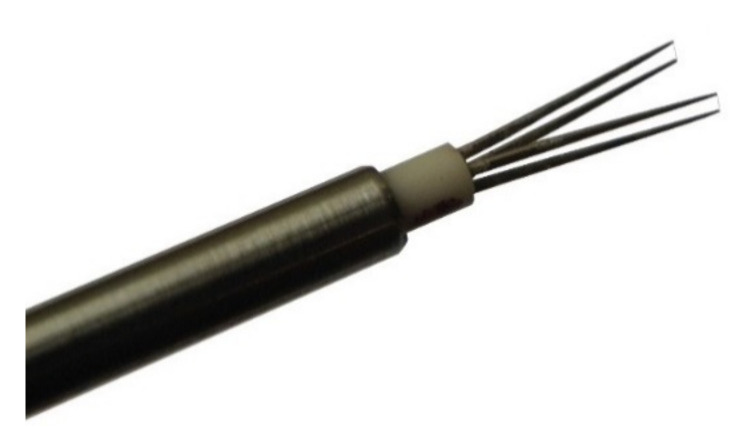
Two-wire measuring sensor.

The presented measurement method uses a two-wire RTD sensor (Figure 1). The 2 mm long wires were placed in the sensor parallel to each other, at a distance of 3 mm. A characteristic and very valuable feature of a hot-wire anemometry wire is its low heat capacity. As a result, it reacts quickly to temperature fluctuations. Since the measurement of the flow velocity using the correlation method consists in determining the time shift between two recorded temperature signals, there is no need to determine the static characteristics of individual temperature sensors in advance, as in the case of classic hot-wire anemometry probes [13,14]. The advantage of this method is also the ability to detect the sense of the velocity vector, which is impossible in case of the conventional hot-wire anemometry probe. The limitation of the presented method is its applicability to measure signals characterized by variability in the temperature range and the dominant component of the flow velocity vector.

In order to measure the flow velocity correctly, this type of sensor must be positioned in the flow so that the both wires are perpendicular to the dominant component of the velocity vector and plane defined by both wires is parallel to the dominant component of the velocity vector. Only then both wires should record the same, time shifted temperature changes.

This method is applicable in various temperature conditions. The temperature limitation of presented sensor is related to the probe resistance. In the current tests, the limit temperature was 350 °C. Such a limit results from the standard construction material used. The presented sensor is equipped with a 5 µm tungsten wire. This allowed for a wide frequency response (approx. 50 kHz). Various materials may be used to increase the resistance of the sensor, but this may change other characteristics such as the bandwidth of the measurement system. The analysis of various models of temperature sensors (wires) is presented in [15].

For the analysis of measurement signals, the correlation function was used, which enables the determination of the similarity of two signals. For discrete signals with a finite number of samples, the correlation can be calculated from the following relation [16]:

(1) $$R_{xy}(k)=\sum_{n=0}^{N-1-\left|k\right|}x(n)y(n-k)$$

where:

*x*(*n*), *y*(*n*)—correlated signals,
*N*—length of the correlated signal section,
*k*—the shift between the signals.

The algorithm presented in this article was based on the fast Fourier transform. This method consists in calculating the Fourier transform for each of the correlated signals (*X*(*k*), *Y*(*k*)), multiplying them with each other (*X*(*k*)·Y*(*k*)), and then determining the inverse transform from the obtained result [16]. Since the applied fast Fourier transform function implements a circular convolution, a zero vector with a length of half the length of the correlated signal was added at the beginning and the end of the signal. In this way, the circular convolution was changed into a linear convolution in which the end of the signal is not changed with the beginning.

In order to limit the value of the zero spectral line in the signal frequency spectrum, its average value was subtracted from the analyzed signal fragment. However, in order to limit the sudden discontinuities at the beginning and the end of the signal, signal weighting was applied in the form of a parametric Tukey window with a 20% transition period (*α*). As a result, the middle part of the signal had the greatest impact on the result during correlation. The Tukey parameter window is determined on the basis of the following dependence:

(2) $$w(i)=\left\{\begin{array}{lll} \frac{1}{2}\left\{1+\cos\left(\pi \left[\frac{2i}{\alpha \left(M-1\right)}-1\right]\right)\right\} & \mathrm{for} & 0\leq i\leq \frac{\alpha \left(M-1\right)}{2}\\ 1 & \mathrm{for} & \frac{\alpha \left(M-1\right)}{2}\leq i\leq \left(1-\frac{\alpha }{2}\right)\left(M-1\right)\\ \frac{1}{2}\left\{1+\cos\left(\pi \left[\frac{2i}{\alpha \left(M-1\right)}-\frac{2}{\alpha }+1\right]\right)\right\} & \mathrm{for} & \left(1-\frac{\alpha }{2}\right)\left(M-1\right)\leq i\leq \left(M-1\right) \end{array}\right.$$

where:

*M*—time window length (s),
*A*—window parameter.

As a result of the algorithm used, the location of the correlation maximum is obtained. On its basis, the time delay *τ* between the signals can be determined:

(3) $$\tau =\frac{x_{\max}}{f},$$

where:

*τ*—time delay (s),
*f*—sampling frequency (Hz),
*x_max_*—shift value determined from the correlation.

**Figure 2:**
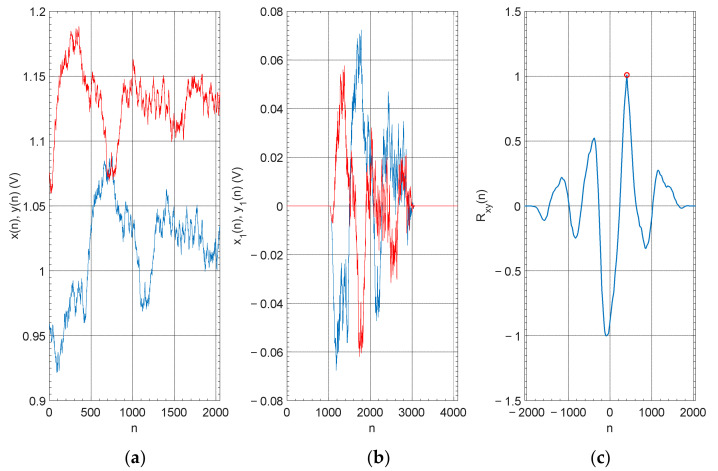
(**a**) The fragments of generated signal, cutting by rectangular window size of 2048 samples. (**b**) The signals prepared to correlation according presented algorithm. (**c**) Correlation matrix with mark correlation maximum.

In order to show how the presented algorithm works, a random signal was generated. In the next stage, this signal was 400 samples shifted. Figure 2a shows a signals fragment, which was cut by a rectangular window size of 2048 samples. Figure 2b shows the same fragments of signal, but after average value subtraction, parametric Tukey window apply and zero vector size of 1024 samples added at the beginning and end of the signal. Such prepared signals were correlated. As a result, a correlation matrix was received (Figure 2c). The position of the element with the maximum value in the correlation matrix is related to the shift of the signal.

Basing on the time delay and the distance between the sensor wires, the flow velocity of the tested medium can be determined:

(4) $$v=\frac{d}{\tau },$$

where:

*v*—measured speed (ms^−1^),
*d*—wire distance (m).

It should be remembered that the applied calculation method enables correct velocity measurement only for the flow direction that is parallel to the plane formed by the wires. In the case of other flow directions, the result of the measurement will be the projection of the flow velocity vector onto the plane formed by the wires.

The results of the presented measurement algorithm are influenced by the following parameters: medium speed, sampling frequency, window length and the distance between the sensor wires.

**Figure 3:**
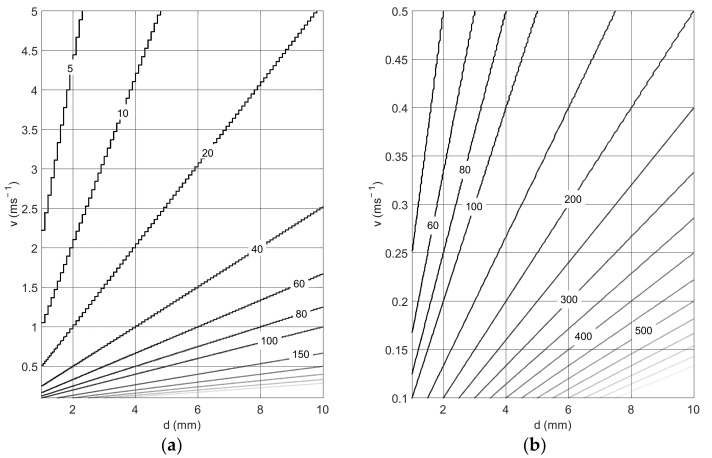
The value of the shift of the correlation maximum depending on the distance *d* between the wires and on the flow velocity *v*, for a sampling frequency of 10 kHz and velocity in the range (**a**) 0.1 to 5 ms^−1^ and (**b**) 0.1 to 0.5 ms^−1^.

Figure 3 shows the size of the shift (expressed in samples) by means of isolines, as a function of distance d between the wires in the measuring sensor and the value of the flow velocity v for a sampling frequency of 10 kHz. The calculations were made for the wires spacing 1 to 10 mm and the velocity values 0.1 to 5 ms^−1^. Figure 3b shows a graph for low velocities: 0.1 ms^−1^ up to 0.5 ms^−1^. The increase in the flow velocity of the medium causes the number of samples responsible for the displacement to decrease. In the case of very small distances between the wires, these are very small shifts. Such small shifts of the real signals may be difficult to determine correctly by the algorithm. Therefore, they will cause large errors in determining the velocity value. Therefore, it is very important to select the appropriate wire spacing for the tested flow.

**Figure 4:**
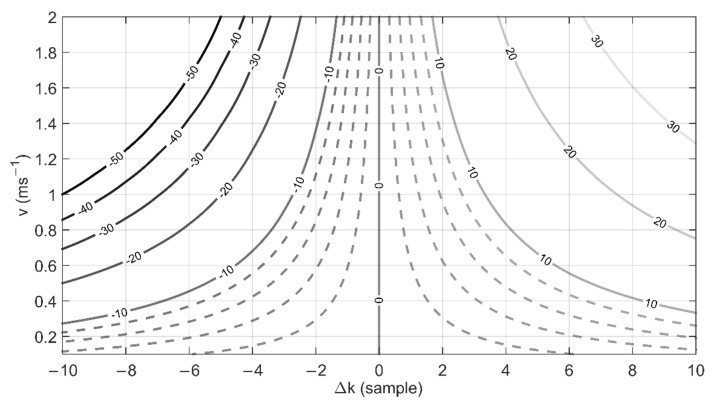
Relative error in determining the flow velocity as a function of the uncertainty of the line shift Δ*k* and the set velocity *v*.

In order to show the influence of the erroneous determination of the maximum shift on the obtained results, Figure 4 presents the percentage relative error of the determined velocity in the case where the measurement algorithm incorrectly determines the position of the correlation maximum. The calculations were made for a sensor with wires placed at a distance of 3 mm from each other, sampling frequency 10 kHz and velocities in the range of 0.1 ms^−1^ to 2 ms^−1^. In the case of low velocity values, the uncertainty of estimating one line ±1 will translate into the velocity determination error lower than 2%. In the case of uncertainty of estimation of the spectral line ±10, this error increases to 6%. For higher velocity values, the relative error increases from about 6% for the uncertainty of estimating one line by ±1 sample, to over 40% for the uncertainty of estimating the line by ±10 samples.

## 3. Testing the Correlation Method of Velocity Measurement

**Figure 5:**
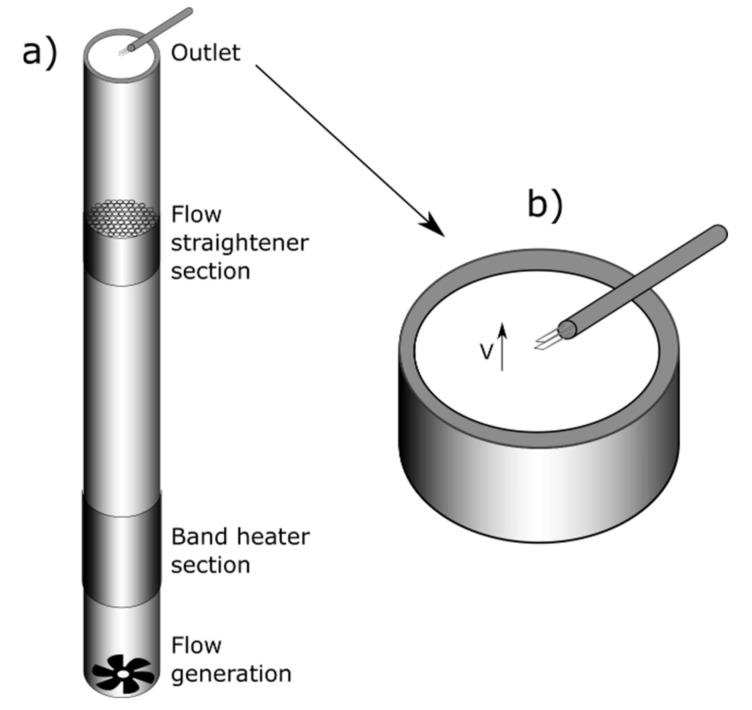
(**a**) Scheme of the measuring stand for setting the flow of variable temperature and velocity. (**b**) Position of the sensor during measurements.

The presented measurement method was tested on a laboratory stand that allows for setting a flow of variable temperature and velocity (Figure 5a). The sensor was fixed so that both the wires and the probe support were in line with the flow direction (Figure 5b). During the experiment, the flow was generated using an inverter-controlled side channel fan. A variable temperature signal was obtained with the use of free convection, the source of which were band heaters placed along the pipe constituting the heating chamber [17]. In this way, the flow with the dominant vertical velocity component was obtained. The measurements were carried out for four velocity values: 0.5, 0.75, 1 and 2 ms^−1^. The sampling frequency was 10 kHz.

**Figure 6:**
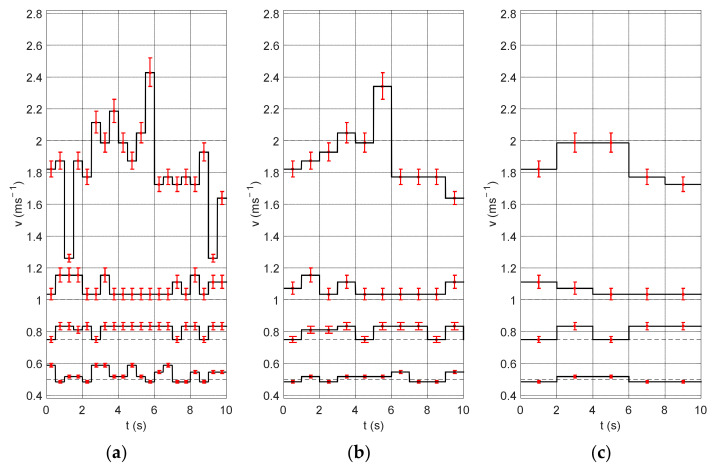
Cumulative results of the first experiment obtained for four velocity values: 0.5, 0.75, 1, and 2 ms^−1^ and different measurement windows: (**a**) 0.5 s, (**b**) 1 s, and (**c**) 2 s.

Figure 6 shows the experiment results obtained for different measurement window lengths (0.5, 1, 2 s). Changing the length of the measurement window influences the time resolution, thanks to which speed fluctuations can be distinguished with smaller windows. Short measurement windows bring out the details of the signal. On the other hand, they cannot be too small, so that the algorithm would detect low flow velocity. Additionally, the graphs show the uncertainty of the method, i.e., the velocity value determined for the shift of the correlation maximum by ±1 sample. It is clearly visible that this error increases with the velocity value. This is due to the fact that for the higher velocity values the shift is smaller and therefore the shift of the maximum position by one sample has a greater influence on the obtained results (this relation was visible in Figure 4).

**Figure 7:**
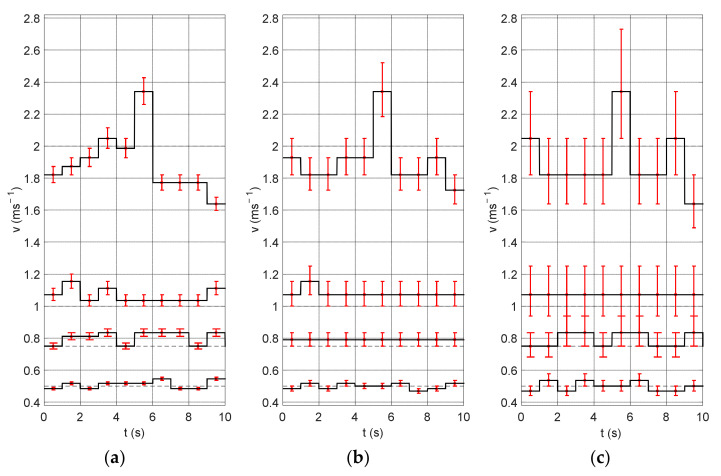
Cumulative results of the experiment for different sampling frequencies: (**a**) 10 kHz, (**b**) 5 kHz and (**c**) 2.5 kHz.

Figure 7 shows the same signal fragments for a 1-s measurement window and three different sampling frequencies: 10, 5 and 2.5 kHz. Lower sampling frequencies were obtained by decimating the signal by two and four times. It can be seen that in the case of low frequency the obtained amplitude resolution is much lower and that the measurement uncertainties are very high. In practice, the measurement uncertainties make it impossible to correctly determine the velocity changes occurring in the signal.

A quantitative analysis was carried out for the experiments performed. The following indicators were determined: average velocity value (*v_avg_*), minimum velocity value (*v_min_*), maximum velocity value (*v_max_*), range (difference between the smallest and the largest value of velocity), standard deviation (*σ*), and mean square error (*ε*), calculated from Equation (5):

(5) $$\varepsilon =\frac{1}{m}\overset{m-1}{\underset{i=0}{\sum }}{\left(v_{i}-v_{p}\right)}^{2}$$

where:

*v_i_*—velocity obtained for *i*th measurement window (ms^−1^),
*v_p_*—preset velocity value (ms^−1^),
*m*—number of calculated velocities.

The obtained results are presented in Table 1 (for the data from Figure 6) and Table 2 (for the data from Figure 7). On the basis of the obtained statistical indicators, it is not possible to clearly determine, which set of algorithm parameters (length of measurement window, frequency) gives the best results. Increasing the window or decreasing the sampling rate leads to lower mean square error values. This is because the resolution of the method is decreased. Part of the fluctuations appearing in the signal is averaged by using too large a measurement window or lost when the sampling frequency is too low. On the other hand, reducing the measurement window or increasing the sampling frequency brings out the fluctuations in the signal and thus increases the mean square error. The obtained standard deviation for our experiments are similar to the standard deviation, which we obtained, when velocity of the reference flow profile was analyzed.

**Figure 8:**
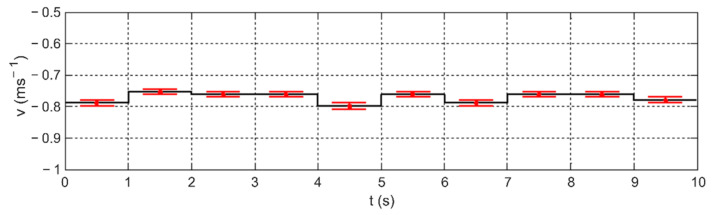
The results of the method sensitivity test to the change of the flow direction on the second measuring wire (negative sense of the velocity vector).

In another experiment, the sensor was rotated 180° in relation to its probe support. Figure 8 shows the results obtained for such arrangement of the sensor. The influx on the second measuring wire resulted in negative velocity values. In this way, it was shown that the presented measurement method is sensitive to the change of the flow direction.

## 4. Modification of the Measurement Algorithm

**Figure 9:**
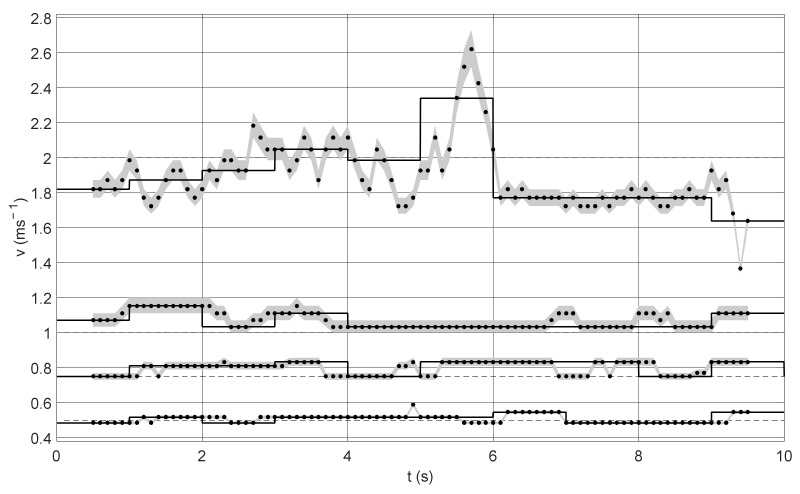
Results obtained after modification of the measurement algorithm: black dots of velocity values obtained for a measurement window with a length of 1 s, shifted every 0.1 s, grey color represents the measurement uncertainty, solid line represents results obtained for a measurement window with a length of 1 s, shifted every 1 s.

In order to improve the quality of the measurement algorithm, the measurement window may be moved by a section of its length. Figure 9 shows the results for the first of the presented experiments (Figure 6b) with a 1 s long measurement window shifted every 0.1 s. The black dots show the individual velocity values obtained in this way. The measurement uncertainty is marked in grey. The black line presents the velocity values with the measurement window shifted by its entire length.

Shifting of the measurement window every 0.1 s increased the measurement time resolution, without the need to reduce the length of the measurement window. As a result, there is no risk of not detecting very low velocities, and at the same time, it is possible to detect fluctuations in flow velocity. The above possibilities are paid for by a significant increase in computational costs. Gross errors were also identified in the obtained results (for 0.5 and 2 ms^−1^). Such errors are not included in the further analysis.

Determination of quantitative parameters was also carried out for the modification of the measurement algorithm. The obtained results are presented in Table 3. Shifting the window every 0.1 s caused additional fluctuations of the measured velocity, without significantly changing the errors. 

## 5. Conclusions

The method of measuring the flow velocity under non-isothermal conditions was tested in the flow with the dominant component of the flow velocity vector. As part of the conducted experiments, the method was verified in terms of sensitivity to changes of the sense and flow velocity values. The conducted research has shown that, using the presented method, it is possible to measure the velocity in non-isothermal flows, with the use of temperature fluctuations as a marker. This method allows for the verification of numerical models in the field of heat and mass transfer [18]. The scope of the method’s applications is limited only by the temperature resistant range of the sensors used and their transmission band. The main advantages of the method are its insensitivity to changes in the static characteristics of thermometric sensors, which means that there is no need to calibrate the sensors. The measuring range and time resolution of the obtained results can be adjusted by selecting the appropriate sampling frequency (taking into account the dynamic properties of the sensors) and the length of the window.

## Figures and Tables

**Table 1:** Qualitative indicators obtained for four velocity values (*v*) and different length of measurement windows (*M*).

*M* (s)	*v* (ms^−1^)	*v_avg_* (ms^−1^)	*v_min_* (ms^−1^)	*v_max_* (ms^−1^)	Range (ms^−1^)	*ε*	*σ*
0.5	0.50	0.531	0.484	0.588	0.104	0.003	0.041
1.0	0.50	0.510	0.484	0.546	0.062	6.36 × 10^−4^	0.025
2.0	0.50	0.497	0.484	0.517	0.033	2.75 × 10^−4^	0.018
0.5	0.75	0.816	0.750	0.833	0.083	0.005	0.034
1.0	0.75	0.790	0.750	0.833	0.083	0.003	0.040
2.0	0.75	0.800	0.750	0.833	0.083	0.004	0.046
0.5	1.00	1.076	1.035	1.154	0.119	0.009	0.054
1.0	1.00	1.065	1.035	1.154	0.119	0.006	0.044
2.0	1.00	1.057	1.035	1.111	0.077	0.004	0.034
0.5	2.00	1.838	1.260	2.427	1.167	0.096	0.272
1.0	2.00	1.895	1.638	2.341	0.702	0.046	0.197
2.0	2.00	1.858	1.725	1.986	0.261	0.032	0.122

**Table 2:** Qualitative indicators obtained for four velocity values (*v*) and different sampling frequencies (*f*).

*f* (kHz)	*v* (ms^−1^)	*v_avg_* (ms^−1^)	*v_min_* (ms^−1^)	*v_max_* (ms^−1^)	Range (ms^−1^)	*ε*	*σ*
10.0	0.50	0.510	0.484	0.546	0.062	6.36 × 10^−4^	0.025
5.0	0.50	0.499	0.469	0.517	0.048	2.95 × 10^−4^	0.018
2.5	0.50	0.498	0.469	0.536	0.067	7.73 × 10^−4^	0.029
10.0	0.75	0.790	0.750	0.833	0.083	0.003	0.040
5.0	0.75	0.790	0.790	0.790	0.000	0.002	0.000
2.5	0.75	0.778	0.750	0.833	0.083	0.002	0.040
10.0	1.00	1.065	1.035	1.154	0.119	0.006	0.044
5.0	1.00	1.080	1.071	1.154	0.082	0.007	0.026
2.5	1.00	1.071	1.071	1.071	0.000	0.005	0.000
10.0	2.00	1.895	1.638	2.341	0.702	0.046	0.197
5.0	2.00	1.906	1.725	2.341	0.616	0.034	0.168
2.5	2.00	1.900	1.638	2.341	0.702	0.045	0.196

**Table 3:** Qualitative indicators obtained for four velocity values (*v*) and measurement window with length 1 s, shifted 0.1 s and 1 s.

Window Shifted (s)	*v* (ms^−1^)	*v_avg_* (ms^−1^)	*v_min_* (ms^−1^)	*v_max_* (ms^−1^)	Range (ms^−1^)	*ε*	*σ*
0.1	0.50	0.506	0.484	0.546	0.062	4.97 × 10^−4^	0.022
1.0	0.50	0.510	0.484	0.546	0.062	6.36 × 10^−4^	0.025
0.1	0.75	0.798	0.750	0.833	0.083	0.004	0.037
1.0	0.75	0.790	0.750	0.833	0.083	0.003	0.040
0.1	1.00	1.074	1.035	1.154	0.119	0.008	0.046
1.0	1.00	1.065	1.035	1.154	0.119	0.006	0.044
0.1	2.00	1.900	1.638	2.621	0.983	0.042	0.181
1.0	2.00	1.895	1.638	2.341	0.702	0.046	0.197

## Data Availability

The data presented in this study are available on request from the corresponding author. The data are not publicly available due to company’s policy.
